# Associations of Objectively-Assessed Physical Activity and Sedentary Time with Hippocampal Gray Matter Volume in Children with Overweight/Obesity

**DOI:** 10.3390/jcm9041080

**Published:** 2020-04-10

**Authors:** Jairo H. Migueles, Cristina Cadenas-Sanchez, Irene Esteban-Cornejo, Lucia V. Torres-Lopez, Eivind Aadland, Sébastien F. Chastin, Kirk I. Erickson, Andres Catena, Francisco B. Ortega

**Affiliations:** 1 PROFITH “PROmoting FITness and Health through physical activity” Research Group, Sport and Health University Research Institute (iMUDS), Department of Physical Education and Sports, Faculty of Sport Sciences, University of Granada, 18011 Granada, Spain; cristina.cadenas@uca.es (C.C.-S.); ireneesteban@ugr.es (I.E.-C.); luciatl@ugr.es (L.V.T.-L.); ortegaf@ugr.es (F.B.O.); 2 MOVE-IT Research Group, Department of Physical Education, Faculty of Education Sciences, University of Cádiz, 11519 Cádiz, Spain; 3 Biomedical Research and Innovation Institute of Cádiz (INiBICA) Research Unit, Puerta del Mar University Hospital, University of Cádiz, 11009 Cádiz, Spain; 4 Faculty of Education, Arts and Sports, Western Norway University of Applied Sciences, 6851 Sogndal, Norway; Eivind.Aadland@hvl.no; 5 School of Health and Life Science, Glasgow Caledonian University, Glasgow G4 0BA, UK; Sebastien.Chastin@gcu.ac.uk; 6 Department of Movement and Sport Science, Ghent University, 9000 Ghent, Belgium; 7 Department of Psychology, University of Pittsburgh, 3601 Sennott Square, Pittsburgh, PA 15260, USA; kiericks@pitt.edu; 8 Department of Experimental Psychology, Mind, Brain and Behaviour Research Centre (CIMCYC), University of Granada, 18011 Granada, Spain; acatena@ugr.es; 9 Department of Biosciences and Nutrition, Karolinska Institutet, 14183 Huddinge, Sweden

**Keywords:** physical behavior, sedentary behavior, brain health, youth, pre-adolescent

## Abstract

This study investigated physical activity (PA) and sedentary time (SED) in relation to hippocampal gray matter volume (GMV) in pediatric overweight/obesity. Ninety-three children (10 ± 1 year) were classified as overweight, obesity type I, or type II–III. PA was assessed with non-dominant wrist accelerometers. GMV was acquired by magnetic resonance imaging (MRI). Neither PA nor SED associated with GMV in the hippocampus in the whole sample (*p* > 0.05). However, we found some evidence of moderation by weight status (*p* < 0.150). Moderate-to-vigorous PA (MVPA) positively associated with GMV in the right hippocampus in obesity type I (B = 5.62, *p* = 0.017), which remained when considering SED, light PA, and sleep using compositional data (γ = 375.3, *p* = 0.04). Compositional models also depicted a negative association of SED relative to the remaining behaviors with GMV in the right hippocampus in overweight (γ = −1838.4, *p* = 0.038). Reallocating 20 min/day of SED to MVPA was associated with 100 mm^3^ GMV in the right hippocampus in obesity type I. Multivariate pattern analysis showed a negative-to-positive association pattern between PA of increasing intensity and GMV in the right hippocampus in obesity type II–III. Our findings support that reducing SED and increasing MVPA are associated with greater GMV in the right hippocampus in pediatric overweight/obesity. Further studies should corroborate our findings.

## 1. Introduction

Improving brain health during childhood is important to enhance brain development, achieve academic goals, and improve cognition [1]. Within the brain, the hippocampus is crucial for short- and long-term memory [2,3], being a determinant of academic success and cognition in children [4,5]. Furthermore, the hippocampus presents a high degree of plasticity [6,7] (i.e., its capacity to change and reorganize in response to internal and/or external influences) [8]. Among the processes related to this plasticity, neurogenesis and angiogenesis can stimulate changes in the gray matter volume (GMV). GMV in the hippocampus can be amplified by a variety of lifestyle factors [9]; among them, aerobic exercise has been widely investigated [8,10,11]. Aerobic exercise consists in structured and organized physical activity (PA) sessions aimed to improve aerobic fitness. Aerobic fitness is the integrated ability of the organism systems to perform PA, and it is a powerful marker of health in children [12]. Otherwise, PA stands for any movement produced by skeletal muscles which increases the resting energy expenditure [13].

Aerobic fitness is associated with GMV in the hippocampus of children [5,10,14], which makes PA a potential resource to target hippocampal GMV. However, associations of PA with GMV in the hippocampus are inconclusive [14,15]. Herting et al. used a whole-brain approach to test associations between self-reported PA and GMV in 34 male adolescents [15]. Higher self-reported PA was associated with greater GMV in the right pericalcarine, right cuneus, and left precuneus, but it was not associated with GMV in the hippocampus [15]. However, self-report measures of PA are limited because of their low accuracy and social desirability bias, especially in youth [16]. To overcome these limitations, Ruotsalainen et al. used accelerometers to assess PA but found no association with GMV in the hippocampus in 60 adolescents [14]. They reduced the accelerometer data into moderate-to-vigorous PA (MVPA) [14], while other PA intensities remain unstudied. Likewise, sedentary time (SED), defined as awake time spent sitting or reclining with low energy requirement [17], has not been studied in relation to GMV in the hippocampus of children to the best of our knowledge.

Accelerometer-determined SED and PA data have certain features that should be considered. PA is usually monitored for seven days, for which the information is averaged to obtain daily estimates of SED, light PA (LPA), and MVPA together with sleep time [18]. This results in a set of interdependent (i.e., multicollinear) variables as they are constrained to 24 h (i.e., sleep + SED + LPA + MVPA = 24 h). In other words, increasing time in any of these behaviors would reduce the time in at least one of the others, a characteristic usually referred to as ‘closure’ [19,20]. Multicollinearity and closure have not been appropriately handled in previous studies on the association between SED, PA, and GMV in the hippocampus of children [14,15]. Thus, studies using appropriate analytical approaches are needed to study the association between PA and SED with GMV in the hippocampus in children. The rate of hippocampal neurogenesis sharply declines during childhood and continues to decline during adulthood [21]. Therefore, it is crucial to find strategies to stimulate GMV in the hippocampus at young ages to ensure future healthy brains. Promoting PA is a promising strategy which needs further study. 

Previous studies have not specifically investigated the association between SED, PA, and hippocampal GMV in pediatric obesity. Children with overweight/obesity engage in more SED [22], perform less PA [22], and present poorer brain health [23]. Thus, the study of the associations between PA and hippocampal GMV in children with overweight/obesity could provide meaningful information for public health messaging, as well as to appropriately design interventions targeting both physical and brain health in pediatric obesity. Therefore, this study aims to investigate associations of objectively measured SED and PA with GMV in the hippocampus using analytical approaches able to deal with the closed structure and strong multicollinearity of data obtained from accelerometry in children with overweight/obesity. Based on previous research on aerobic exercise [8,10,11], we hypothesized that lower SED and higher PA would associate with greater hippocampal GMV in children with overweight/obesity.

## 2. Material and Methods

### 2.1. Participants and Study Design

We used baseline data from the ActiveBrains project (Identifier: NCT02295072) [24] collected from November 2014 to February 2016 in Granada (Spain). Initially, 110 children enrolled in the ActiveBrains project. Those with valid accelerometer and brain data at baseline were included in this cross-sectional analysis (*n* = 93, 10 ± 1 years of age, 37 girls). More information about the study can be found elsewhere [24]. Briefly, all participants met the inclusion criteria: (1) overweight or obesity based on the World Obesity Federation cut-off points [25,26]; (2) 8–11 years old; (3) no physical disabilities or neurological disorders that affect physical performance; and (4) in the case of females, were not menstruating at the time of the baseline assessment. 

Parents or legal guardians were informed of the purpose of the study and provided written informed consent. The ActiveBrains project was approved by the Ethics Committee on Human Research of the University of Granada.

### 2.2. Accelerometer Data Collection and Processing

Accelerometer data collection and processing criteria are described elsewhere [27,28]. In brief, participants were required to wear accelerometers ActiGraph GT3X+ (ActiGraph, Pensacola, FL, USA) on their non-dominant wrist for 7 consecutive days, and to complete a sleep log with information on time to bed and time out of bed every day. Parents were suggested to supervise their children in the fulfillment of the sleep logs. Accelerometers were initialized to record accelerations at 100 Hz with a dynamic range of ±6 *G*. Raw accelerations were downloaded via the ActiLife v.6.13.3 software (ActiGraph, Pensacola, FL, USA) and processed in the R package GGIR (v.1.5.12) [29,30]. Non-wear time and abnormal high accelerations related to malfunctioning of the accelerometers were imputed by average acceleration during the same time interval from the rest of the days [30]. Sleep time was identified using an automated algorithm guided by the time reported by the participants [31,32]. Finally, SED (<35 m*g*) and intensity-specific PA (LPA: 35–200 m*g*; MVPA: >200 m*g*) were calculated using previously-proposed acceleration thresholds for the non-dominant wrist in children [33,34]. Additionally, the intensity spectrum was defined using time spent in 33 acceleration bands of increasing intensity by 25 m*g* (i.e., time spent in 0–25 m*g*, 25–50 m*g*, 50–75 m*g*, and so on). Only awake time was used to calculate the intensity spectrum variables since sleep and SED can occur at similar acceleration bands, which would confound the interpretation of findings. The average daily values of time spent in each category were calculated as: (weekdays × 5 + weekends × 2) / 7. The participants were excluded if they recorded less than 4 valid days (≥16 h/day), including at least 1 weekend day [18].

### 2.3. Magnetic Resonance Imaging (MRI) Data Acquisition and Processing

All images were collected on a 3.0 Tesla Siemens Magnetom Tim Trio scanner (Siemens Medical Solutions, Erlangen, Germany) with a 32-channel head coil. High-resolution, T1-weighted images were acquired using a 3D MPRAGE (magnetization-prepared rapid gradient-echo) protocol. The acquisition parameters were the following: repetition time = 2300 ms; echo time = 3.1 ms; inversion time = 900 ms; flip angle = 9°; field of view = 256 × 256; acquisition matrix = 320 × 320, 208 slices; resolution = 0.8 × 0.8 × 0.8 mm; and scan duration = 6 min and 34 s. 

Hippocampal volumetric analyses were conducted using FMRIB’s Software Library (FSL) version 5.0.7. (FMRIB analysis group, Oxford, UK). Specifically, we used FMRIB’s Integrated Registration and Segmentation Tool (FIRST) in FSL. FIRST is a semi-automated model-based subcortical segmentation tool which uses the Bayesian framework from shape and appearance models obtained from manually segmented images from the Center for Morphometric Analysis, Massachusetts General Hospital (Boston, MA, USA) [35]. Briefly, FIRST runs a two-stage affine registration to a standard space template (i.e., Montreal Neurological Institute -MNI- space) using 12 degrees of freedom and uses a subcortical mask to exclude voxels outside the subcortical regions. Second, subcortical regions, including the hippocampus, are segmented for both hemispheres separately. Manual volumetric region labels are parameterized as surface meshes and modeled as a point distribution model. In addition, the hippocampus segmentation from FIRST was then split based on the center of gravity of the region into anterior and posterior sub-regions for each hemisphere separately. This resulted in separate anterior and posterior hippocampal segmentation for each participant, for each hemisphere [36,37]. The final segmentations were visually inspected for quality. The volume of each region was obtained from FIRST in mm^3^. 

### 2.4. Confounders

Participants’ body mass, height, peak height velocity, and parental education level were obtained as part of the protocol of the ActiveBrains project [24]. Weight and height were measured twice consecutively with an electronic scale (SECA 861, Hamburg, Germany) and a stadiometer (SECA 225, Hamburg, Germany), respectively, and averaged values were used in analyses. Body mass index (BMI) was calculated as weight (kg) divided by squared height (m^2^). Children were classified as having overweight, obesity type I, and obesity type II–III using the sex- and age-specific BMI cut-offs proposed by the World Obesity Federation [25,26]. Peak height velocity was derived from standing or seated height as a continuous measure of maturational status using the Moore et al. equations: for boys, –8.13 + (0.007 × (age × seated height)); for girls, –7.71 + (0.004 × (age × height)) [38]. Parents reported their highest completed level of education. Parental education level was categorized as both of them, one of them, or neither of them reaching university-level education. Total brain volume was derived from FreeSurfer software version 5.3.0 (Laboratory for Computational Neuroimaging, Athinoula A. Martinos Center for Biomedical Imaging, Harvard Medical School, Boston MA, USA) as the sum of total white matter volume and total GMV.

### 2.5. Statistics

Participants’ descriptive characteristics were summarized as mean and standard deviation (SD) or percentages. Bivariate correlations among PA and SED indicators and between these variables and GMV in the right and left hippocampi were performed. Then, associations of PA and SED (explanatory/independent variables) with GMV in the hippocampus (outcome/dependent variable) were analyzed using different analytical approaches (i.e., multiple standard linear regression using absolute and compositional data and multivariate pattern analysis with absolute data). After testing the potential confounding effect on the associations, the same set of confounders was accounted for in all analyses (i.e., sex, peak height velocity, parental education level, and total brain volume). Interactions between weight status (i.e., overweight, obesity type I, or obesity type II–III) and PA were tested because of the moderator effect shown in previous studies [10,39]. Using multiple linear regression with absolute data, a moderation effect was found in the association of LPA and MVPA with GMV in the right hippocampus (*p* < 0.15). Thus, the analysis was stratified by obesity category. The analytical approaches were implemented as follows.

Multiple linear regression models using absolute PA and SED data were performed to compare associations with previously-published findings. Separate models were performed for each PA intensity and SED. Findings from these models should be interpreted as incrementing time spent in a behavior in isolation (i.e., without considering the remaining behaviors).

Multiple linear regression with compositional data [19,20] was used to study the relative association of PA and SED with GMV in the hippocampus. Compositional data analysis accounts for the relative nature of physical behavior by quantifying the effect of incrementing time in each behavior by reducing the time spent in the rest (i.e., closure). Since time exchange can also occur with sleep time, detected sleep period time (i.e., time from going to bed to waking up) was included in compositional analyses. Isometric log-ratios were firstly calculated and then introduced in multiple linear regression models as previously proposed [19] (see Appendix A for a detailed explanation of the models). Gamma (γ) coefficients with their respective 95% interval inform of the strength and direction of the association. For an accurate estimation of the effect size, isotemporal substitution plots were computed to investigate the effect of increasing LPA and MVPA in the detriment of SED. Findings from compositional models should be interpreted as incrementing time spent in a behavior relative to time spent in the remaining behaviors (or pair-wise time exchange between behaviors in the reallocation plots). 

Multivariate pattern analysis with absolute PA and SED data was used to further understand the associations depicted by previous models. Partial least squares regression was performed since it can handle completely collinear variables through the use of latent modeling [40,41]. Models were cross-validated using Monte Carlo resampling [42] with 1000 repetitions by repeatedly and randomly keeping 50% of the subjects as an external validation set. For each validated partial least squares regression model, a single predictive component was subsequently calculated through target projection [40,43] to express all the predictive variance in the PA intensity spectrum related to GMV in the hippocampus in a single intensity vector. Selectivity ratios with 95% confidence intervals were obtained as the ratio of this explained predictive variance to the total variance for each PA intensity variable [44]; see Appendix B for an in-depth description of selectivity ratio interpretation. Briefly, the selectivity ratio has a range of −1 to 1 and the negative or positive sign informs the direction of the association with the outcome. Associations from the partial least squares regression should be interpreted as each intensity variable’s importance for predicting the outcome, while simultaneously taking into account all intensity bands in one joint model. Thus, the model provides the total association pattern between PA intensity and hippocampal GMV.

All analyses were performed in R (v. 3.6.2), except for the multivariate pattern analysis, which was performed in Sirius v.11.0 (Pattern Recognition Systems AS, Norway).

## 3. Results

Participants’ sociodemographic and anthropometric characteristics, hippocampal GMV, PA, and SED are reported in Table 1. Children spent around 39% of the day in SED, 19% in LPA, and 4% in MVPA, with the remaining 38% spent in bed. SED increased and MVPA decreased with more adverse weight status, while LPA was relatively constant across weight status groups. SED, LPA, and MVPA were correlated in this study sample (r ranging from 0.3 to 0.5, *p* < 0.001; Supplementary Material, Table S1).

**Figure 1:**
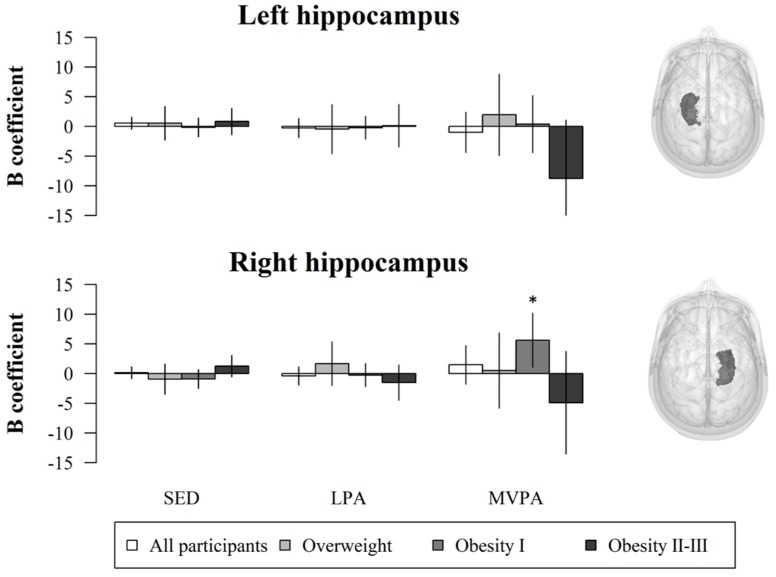
Regression non-standardized B coefficients and 95% confidence intervals (i.e., error bars) for the association of SED, LPA, and MVPA with GMV in the hippocampus adjusted for sex, peak height velocity, parental university level, and total brain volume. *Indicates statistical significance (*p* < 0.05). SED: sedentary time; LPA: light physical activity; MVPA: moderate-to-vigorous physical activity.

Bivariate correlations of PA and SED with GMV in the left and right hippocampi stratified by weight status are presented in the supplementary material (Table S2). Non-standardized beta coefficients with their respective 95% confidence intervals from the multiple linear regression models with absolute PA and SED data are shown in Figure 1. Overall, neither SED nor PA were associated with GMV in the left or right hippocampi in the whole study sample (*n* = 93, *p* > 0.05). Separate analyses in weight status groups depicted that MVPA was positively associated with GMV in the right hippocampus in children with obesity type I (*n* = 41, *p* = 0.017). 

**Figure 2:**
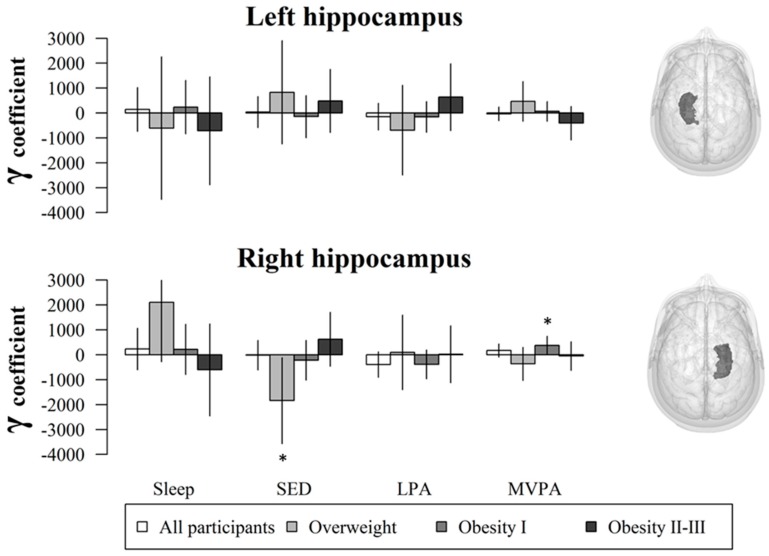
Compositional models γ coefficients and 95% confidence intervals (i.e., error bars) for the association of SED, LPA, and MVPA with GMV in the hippocampus adjusted for sex, peak height velocity, parental university level, and total brain volume. Each bar represents the association of the pertinent behavior (e.g., SED) relative to the remaining behaviors (e.g., LPA, MVPA, and sleep) with GMV in the hippocampus. * Indicates statistical significance *(p* < 0.05). SED: sedentary time; LPA: light physical activity; MVPA: moderate-to-vigorous physical activity.

Figure 2 shows γ coefficients from compositional models with their respective 95% confidence intervals. The γ coefficients represent the direction and strength of association between the isometric log-ratio (this is, the association of each behavior relative to the remaining behaviors) and GMV in the left and right hippocampi. Consistent with the standard multiple regression models, SED and PA were not associated with either left or right hippocampi in the whole sample (*n* = 93, *p* > 0.05). The association of MVPA relative to SED, LPA, and sleep with GMV in the right hippocampus was significant in the sub-sample of children with obesity type I (*n* = 41, *p* = 0.040). Likewise, SED relative to LPA, MVPA, and sleep was negatively associated with GMV in the right hippocampus in children with overweight (*n* = 23, *p* = 0.038). MVPA was not associated with GMV in the sub-sample of children with obesity type II–III using compositional models. 

**Figure 3:**
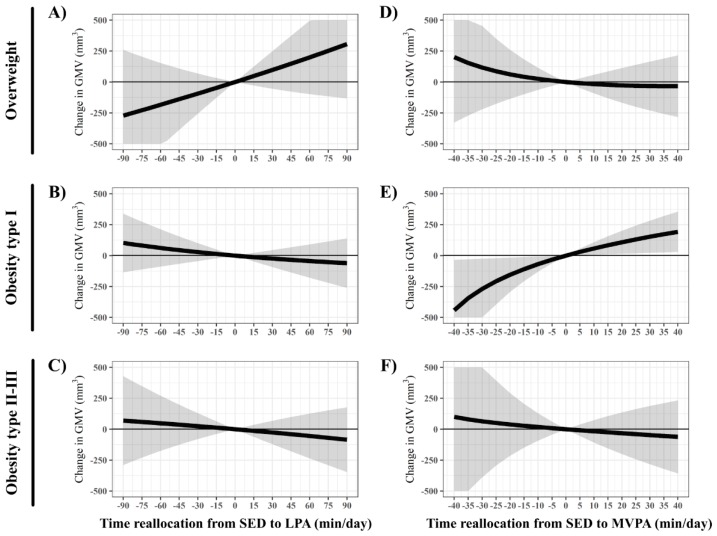
Effect of reallocating time from SED to LPA (Panels **A**–**C**) and to MVPA (Panels **D**–**F**) on the association with gray matter volume in the right hippocampus using compositional models. SED: sedentary time; LPA: light physical activity; MVPA: moderate-to-vigorous physical activity.

The hypothetical effect of increasing either LPA or MVPA in the detriment of SED on GMV in the right hippocampus is presented in Figure 3. The subsample of children with obesity type I presented a significant positive effect of reallocating time from SED into MVPA on GMV in the right hippocampus. Since neither SED nor PA were associated with GMV in the left hippocampus (Figure 2), isotemporal reallocations were not depicted for this region. 

**Figure 4:**
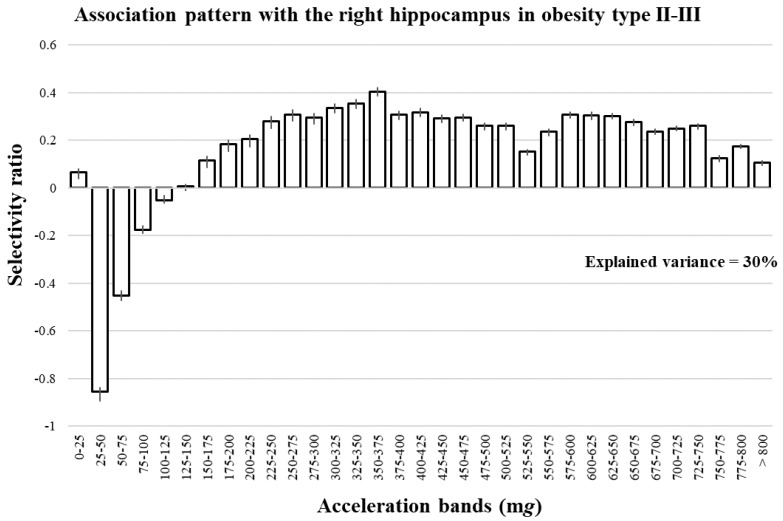
Association pattern of the physical activity spectrum with gray matter volume in the right hippocampus in children with obesity type II–III.

Finally, a multivariate pattern analysis with partial least squares regression was performed to investigate the association of the absolute PA pattern with GMV in the hippocampus. Similar to previous analyses, the PA pattern was not associated with GMV in the hippocampus in the whole sample. Regarding the stratified analyses for weight status, we found that the absolute PA pattern was associated with GMV in the right hippocampus in those children with obesity type II–III (Figure 4). Negative selectivity ratios were found with low acceleration bands (representative of SED and LPA), while positive selectivity ratios were observed in high acceleration bands (indicators of MVPA). The most negative association was found in the acceleration band of 25–50 m*g*, which is an indicator of SED (selectivity ratio = −0.855, which means this band explains ~85% of the 30% explained by the latent components, i.e., ~25%), while the most positive was found in the 350–375 m*g* band, an indicator of moderate PA (selectivity ratio = 0.404). No associations were found in other weight groups with the left or the right hippocampi using multivariate pattern analysis.

## 4. Discussion

The main finding of this study was the lack of association between SED, LPA, and MVPA with hippocampal GMV in children with overweight/obesity. This lack of association persisted after performing the compositional data analysis and multivariate pattern analysis models, which take into account the relative nature and closure characteristics of the accelerometer-determined SED and PA data. SED, LPA, and MVPA were correlated in this study sample, which confirms our decisions on using analytical approaches to handle this co-dependency. Further studies using these analytical approaches will corroborate our findings. Nonetheless, we found that associations were potentially moderated by weight status, which could be hiding any association in certain weight groups; thus, we performed separate analyses for weight status categories (i.e., overweight, obesity type I, and obesity type II–III). In this regard, we found a positive association of MVPA with GMV in the right hippocampus in children with obesity type I (using multiple regression with standard and compositional data) and in obesity type II–III (using multivariate pattern analysis). Likewise, we found that longer time in SED relative to LPA, MVPA, and sleep was associated with lower GMV in the right hippocampus in children with overweight (only in compositional data models). Otherwise, neither of the analyses performed depicted significant associations between PA or SED with GMV in the left hippocampus.

Relative to the moderation effect by weight status, it should be considered that our sample sizes in each subgroup are limited and these findings should be cautiously interpreted. We used the World Obesity Federation categories [25,26] because: (1) they have been extensively related to both physical [45] and brain health [39]; and (2) these cut-off points were developed as sex- and age-specific in pediatric ages to connect at the age of 18 years with the adults BMI worldwide accepted cut-off points (i.e., 25 for overweight, 30 for obesity type I, and ≥35 for obesity type II–III). A previous study described a moderation effect of weight status on the acute effects of walking on memory in children [46]. Specifically, they found a single bout of walking to be effective in children with overweight/obesity to substantially improve word recognition memory performance, while it was not effective in children with normal weight [46]. The authors proposed circulating inflammatory markers to be tested as responsible for this moderation effect. In brief, obesity is characterized by an unhealthy inflammatory response and PA has demonstrated higher anti-inflammatory and neuroprotective effects in obesity-induced brain inflammation [47,48,49,50,51]. Based on this, it would be expected that a larger association of PA with GMV in the hippocampus as the weight status is worse, but we did not find this linear trend. In this regard, further studies with larger sample sizes should deeply study this moderation effect with larger sample sizes.

Relative to our separate analyses for weight status groups, PA appears to be positively associated with GMV in the right hippocampus. Equally significant, we found that using appropriate analytical approaches to account for the data singularities of accelerometer-determined PA (i.e., closure and multicollinearity) is needed to elucidate the pattern of associations. In this regard, it appeared to be a negative non-adjusted association between MVPA and GMV in the left hippocampus in obesity type II–III (Table S2), which disappeared in compositional models and turned positive in multivariate pattern analysis. Since sample sizes were relatively small in our analyses, we could be under-powered to detect small-to-medium associations, so there is a risk of spurious associations in our findings. Therefore, further appropriately-powered studies should corroborate and extend our findings. The associations differed between analytical approaches. The compositional data analysis found an association with obesity type I and the multivariate pattern analysis with obesity type II–III. In this regard, compositional analysis is interpreted in terms of increasing a behavior in exchange with others; thus, we found that increasing MVPA relative to decreases in SED, LPA, and sleep was positively associated with GMV in the right hippocampus in obesity type I. Otherwise, the multivariate pattern analysis is interpreted in terms of absolute changes in a certain behavior fully considering multicollinearity among PA intensities. In this sense, we found that MVPA is positively associated with GMV in the right hippocampus in obesity type II–III. Our models with compositional data required the inclusion of three extra covariates to account for the relative nature of the data, which could imply that even larger sample sizes are needed to investigate associations with compositional models. The multivariate pattern analysis fully considers multicollinearity among PA variables and it is less affected by sample size, which could explain why the MVPA association was found using this approach in obesity type II–III even with its limited sample size. We do not have a large enough sample size to elucidate why associations differed across analytical approaches; thus, we suggest considering these associations with caution. Additionally, we call for further studies with larger sample sizes to apply these analytical approaches (i.e., compositional data analysis and multivariate pattern analysis) that are more suitable than standard linear regression to accelerometer-determined PA and SED data.

Hippocampal plasticity across the lifespan has been previously confirmed [6,7]. However, the rate of hippocampal neurogenesis sharply declines during childhood and continues to decline during adulthood [21]. Thus, it is crucial to find strategies to stimulate hippocampal plasticity at young ages to ensure future healthy brains. Aerobic fitness is among the factors associated with hippocampal GMV [5,10,14], which makes PA a potential resource to target hippocampal GMV. In this study, we found lower SED and higher MVPA to be associated with the GMV in the right hippocampus in children with overweight, but no associations were found for the left hippocampus. Although both left and right hippocampi are related to episodic memory in humans, they have differential functions with the left being involved in verbal and linguistic memory and the right in non-verbal and visuospatial memory [52,53]. Hippocampal structures do not follow similar maturational trajectories [54]. It is plausible that the left and the right hippocampi show differential plasticity, especially in youths’ developing brains, which could explain why we found associations only with the right hippocampus. Therefore, it seems that reducing SED and incrementing the time devoted to PA may be advised to stimulate higher GMV in the hippocampus in children with obesity type I. However, since we cannot conclude that a causal relationship exists, further randomized controlled trials that are appropriately powered to test the moderating role of weight status should be carried out. 

We decided to focus our analyses on the hippocampus given that it is a brain region highly sensitive to PA in older populations [36,55]. Thus far, evidence in youths is limited with only two previous studies investigating the relationship of PA with GMV in adolescents [14,15]. None of these studies directly focused on the hippocampus, but rather used a whole-brain approach [15] or regional analyses [14] and did not find associations between PA and GMV in the hippocampus. These studies presented several limitations such as the use of self-reported tools to estimate PA [15], the reduction of PA data into one single variable (i.e., MVPA), hardly representative of the whole PA pattern [14,15], and the use of standard analytical approaches to test associations without considering the singularities of PA data (i.e., closure and multicollinearity) [14,15]. Furthermore, both studies had relatively small sample sizes (i.e., 34 and 60 participants) and were focused on adolescence, a period in which hippocampal neurogenesis might not be sensitive to external factors [21], such as PA or SED. This study overcomes previous limitations by using accelerometers to estimate PA, SED, and sleep. Considering the singularities of PA data with appropriate analytical approaches [19,41] applied in a sample of nearly a hundred children, our findings support the general public health recommendations on reducing SED and increasing PA to benefit brain health, specifically GMV in the right hippocampus. The overweight/obesity condition of our sample is important since these children usually have poorer physical and brain health profiles [23], thus, the study of potential interventions to improve their health status is a global need. In this regard, and similar to some previous studies [10,39], we found a potential moderator effect of weight status on the association between PA and GMV in the hippocampus that should be further corroborated with larger sample sizes. No less important is the fact that MVPA was associated in obesity type I using compositional analysis, and in obesity type II using multivariate pattern analysis. Although a moderation effect of weight status has been previously reported [10,39], it would be expected that the magnitude of the association increases as does the weight status [46]. The lack of this increasing size of the association could be partly explained because of our limited sample size (*n* = 23 and 29, respectively), which should be corroborated with further well-powered studies. Although previous studies failed at finding an association between PA and GMV in children [14,15], the positive association of aerobic fitness and GMV in several brain regions (including the hippocampus) has been widely reported in children [5,10,14]. Aerobic fitness could be an indicator of PA level since it is linearly associated with MVPA (standardized β around 0.3–0.4, *p* < 0.01 in this specific sample) [56]; however, the direct study of the behavior (i.e., PA and SED) is important for public health for various reasons: first, aerobic fitness is partially explained by genetic factors, which are not modifiable by PA; second, although PA is effective at improving aerobic fitness, there could be other physiological changes related to health behavior (PA) but not to aerobic fitness; third, the interpretation and applicability of aerobic fitness to public health is not straightforward (i.e., the general population is not familiar with the interpretation of aerobic fitness values, nor with the strategies that should be followed to increase aerobic fitness); and fourth, in contrast, knowledge on how much time should be spent in certain activity types/intensities to improve brain health is more easily understandable by the general population. As an example, in our sub-sample of children with obesity type I, reallocating 20 min/day from SED to MVPA was associated with 100 mm^3^ (3%) increase in gray matter in the right hippocampus. 

The main limitations of this study were: the cross-sectional design of the study, which does not allow causal interpretation of findings; although our study involves a larger sample size than previous studies, even more powerful studies are needed to confirm or contrast our findings; and sample sizes in the weight groups were asymmetric. We could have used the median split or terciles to match sample sizes but decided to use evidence-based and standard cut-offs. On the other hand, strengths of this study include: the use of accelerometers to objectively assess PA, SED, and sleep; the inclusion of sleep in compositional models to test its relative effect on the associations of PA and SED with GMV in the hippocampus; the use of MRI for the quantification of GMV in the hippocampus; the use of modern analytical approaches to analyze accelerometer data, which allows appropriate conclusions by handling the PA data singularities (i.e., closure and multicollinearity); and the focus on children with overweight/obesity, which is a harmful condition for both physical and brain health in children.

## 5. Conclusions

Our findings indicate that PA and SED were not associated with GMV in the hippocampus in children with overweight/obesity. However, we found some evidence of moderation by weight status in the associations, so that reducing SED and engaging in more MVPA were associated with greater GMV in the right hippocampus. Specifically, reallocating 20 min/day from SED to MVPA would be associated with 100 mm^3^ more GMV in the right hippocampus in children with obesity type I. We performed an in-depth data analysis by using compositional data and multivariate pattern analysis on accelerometer-determined PA data. These findings should be further confirmed by future studies.

## Figures and Tables

**Table 1:** Descriptive characteristics of participants.

	All (*n* = 93, 37 girls)	Overweight (*n* = 23, 9 girls)	Obesity I (*n* = 41, 15 girls)	Obesity II–III (*n* = 29, 13 girls)
Age (years)	10.01 (1.12)	10.13 (1.08)	10.29 (1.04)	9.51 (1.14)
PHV (years)	–2.31 (0.97)	–2.36 (1.04)	–2.1 (0.93)	–2.58 (0.91)
Weight (kg)	55.67 (10.69)	46.32 (7.30)	56.92 (9.63)	61.74 (9.31)
Height (cm)	143.95 (8.10)	142.16 (8.80)	146.59 (7.78)	141.84 (7.08)
BMI (kg/m^2^)	26.74 (3.63)	22.64 (1.41)	26.26 (2.06)	30.68 (2.36)
Total brain volume (mm^3^)	1202.07 (106.67)	1210.02 (99.41)	1221.03 (94.93)	1169.54 (122.50)
Parental university level, %				
Neither parent	68	57	59	90
One parent	16	17	22	7
Both parents	16	26	19	3
Gray matter volume				
L hippocampus (mm^3^)	3468.73 (371.48)	3387.17 (348.96)	3572.49 (346.4)	3386.71 (397.67)
R hippocampus (mm^3^)	3597.99 (382.49)	3568.46 (420.35)	3709.9 (354.77)	3463.19 (352.41)
Physical activity				
SED (min/day)	561.39 (60.85)	534.25 (71.12)	559.35 (50.59)	585.78 (57.52)
LPA (min/day)	275.36 (39.75)	277.85 (40.42)	273.16 (43.31)	276.49 (34.83)
MVPA (min/day)	54.61 (20.91)	61.76 (26.79)	53.84 (19.66)	50.05 (16)

Data are presented as mean (standard deviation) or percentages. PHV: peak height velocity; BMI: body mass index; L: left; R: right; SED: sedentary time; LPA: light physical activity; MVPA: moderate-to-vigorous physical activity.

## Supplementary Materials

The following are available online at https://www.mdpi.com/2077-0383/9/4/1080/s1, Table S1: Bivariate correlations between PA and SED variables, Table S2: Bivariate correlations between PA, SED, and GMV in the left and right hippocampi for the whole sample and stratified by weight status.

## Appendix A

### Compositional Data Transformation

We used the compositional data analysis to study the association of the time-use composition (i.e., SED, LPA, MVPA, and sleep) with GMV in the hippocampus. This model uses the isometric log-ratio (ILR) coordinates of the time-use composition along with confounders as explanatory variables; and the GMV in either the left or the right hippocampus as the response variable. Each ILR component consisted of three coordinates:

(A1) zSB=(z1:34lnSB(LIPA·MVPA·Sleep)13, z2:23lnLIPA(MVPA·Sleep)12, z3:23lnMVPASleep)

(A2) zLIPA=(z1:34lnLIPA(MVPA·Sleep·SB)13, z2:23lnMVPA(Sleep·SB)12, z3:23lnSleepSB)

(A3) zMVPA=(z1:34lnMVPA(Sleep·SB·LIPA)13, z2:23lnSleep(SB·LIPA)12, z3:23lnSBLIPA)

(A4) zSleep=(z1:34lnSleep(SB·LIPA·MVPA)13, z2:23lnSB(LIPA·MVPA)12, z3:23lnLIPAMVPA)

The compositional regression coefficient (*γ*) of each ILR component represents the direction and strength of the association relative to other behaviors. For example, *γ* > 0 for z_1_ in Equation (A1) would be interpreted as SB relative to LIPA, MVPA, and sleep as positively associated with GMV in the hippocampus. Likewise, 95% confidence intervals (CIs) and *p*-values for the *γ* were calculated using a likelihood ratio test [57]. Since we aim to investigate the association of each behavior relative to the remaining behaviors, we report estimates from the z_1_ coordinate of the equations above. Isotemporal substitution plots were drawn to estimate the effect size of the association resulting from reallocating time from SED into either LPA or MVPA. Isotemporal substitution models with compositional data [57] were used to calculate the expected HRs associated with the range of compositions arising from two-way reallocations of time between behaviors. The compositional mean was calculated and used as the reference.

## Appendix B

### Interpretation of Selectivity Ratio in Multivariate Pattern Analysis

The selectivity ratio is a statistic which tries to separate important from irrelevant information. Applied to accelerometer-determined physical activity data, the selectivity ratio tries to identify those acceleration bands which are relevant and associate to a given outcome (in this case, GMV in the right hippocampus). Thus, the selectivity ratio used in this study represents the ratio of the explained variance to the total explained variance of the outcome. That is, the intensity spectrum shown in Figure 4 explains 30% of the variance in the GMV in the right hippocampus, which would be the total variance explained. This 30% explanation comes from the latent components extracted using partial least squares regression and validated with the Monte Carlo resampling. Then, the selectivity ratio for each acceleration band informs the percentage of the variation in this 30% which is explained by the pertinent acceleration band. For example, the 25–50 m*g* acceleration band shows a selectivity ratio of −0.855, which means this band explains ~85% of the 30% explained by the latent components (i.e., ~25% of the variance in the outcome). The selectivity ratio has a range of −1 to 1 and the negative or positive sign only informs the direction of the association with the outcome.

To note whether the selectivity ratio provides the percentage of explanation of each acceleration band out of the total variation explained in the outcome, it could be expected that all selectivity ratios sum up to 1 (i.e., 100%). The reason why our selectivity ratios do not sum up to 1 (Figure 4) is that the explanatory variables are not independent of each other (as they should be in standard analytical methods). Thus, multivariate pattern analysis does not inform their separate association with the outcome, which is reasonable given their strong interrelationships (i.e., multicollinearity). Furthermore, given the closure in PA and SED data, meaningful interpretations should incorporate reallocation of time instead of separate associations as a basis for interpretation [19,58]. To illustrate this concept, we performed the compositional data analysis with the traditional physical behavior variables (i.e., SED, LPA, MVPA, and sleep).
