# Supplementary material for: Associations of Objectively-Assessed Physical Activity and Sedentary Time with Hippocampal Gray Matter Volume in Children with Overweight/Obesity
Source: J Clin Med. 2020 Apr 10;9(4):1080. doi: 10.3390/jcm9041080 (PMC7231303; doi:10.3390/jcm9041080)
Supplement: Supplementary file 1 [file jcm-09-01080-s001.pdf]

Supplementary Materials: Associations of Objectively-assessed Physical Activity and Sedentary Time with Hippocampal Gray Matter Volume in Children with Overweight/Obesity

Jairo H. Migueles [1,*], Cristina Cadenas-Sanchez [1,2,3], Irene Esteban-Cornejo [1], Lucia V. Torres-Lopez [1], Eivind Aadland [4], Sébastien F. Chastin [5,6], Kirk I. Erickson [7], Andres Catena [8] and Francisco B. Ortega [1,9]

1 PROFITH "PROmoting FITness and Health through physical activity" Research Group, Sport and Health University Research Institute (iMUDS), Department of Physical Education and Sports, Faculty of Sport Sciences, University of Granada, 18011 Granada, Spain; cristina.cadenas@uca.es (C.C.-S.); ireneesteban@ugr.es (I.E.-C.); luciatl@ugr.es (L.V.T.-L.); ortegaf@ugr.es (F.B.O.)
2 MOVE-IT Research Group, Department of Physical Education, Faculty of Education Sciences, University of Cádiz, 11519 Cádiz, Spain.
3 Biomedical Research and Innovation Institute of Cádiz (INiBICA) Research Unit, Puerta del Mar University Hospital, University of Cádiz, 11009 Cádiz, Spain.
4 Faculty of Education, Arts and Sports, Western Norway University of Applied Sciences, 6851 Sogndal, Norway; Eivind.Aadland@hvl.no (E.A.)
5 School of Health and Life Science, Glasgow Caledonian University, G4 0BA Glasgow, UK; Sebastien.Chastin@gcu.ac.uk (S.F.C)
6 Department of Movement and Sport Science, Ghent University, 9000 Ghent, Belgium.
7 Department of Psychology, University of Pittsburgh, 3601 Sennott Square, Pittsburgh, PA, USA; kiericks@pitt.edu (K.I.E.)
8 Department of Experimental Psychology, Mind, Brain and Behaviour Research Centre (CIMCYC), University of Granada, 18011 Granada, Spain; acatena@ugr.es (A.C.)
9 Department of Biosciences and Nutrition, Karolinska Institutet, 14183 Huddinge, Sweden.
* Correspondence: jairohm@ugr.es

Table S1. Bivariate correlations between PA and SED variables.

		SED	LPA	MVPA
SED	r	-	-0.443	-0.510
	P	-	<0.001	<0.001
LPA	r	-0.443	-	0.337
	P	<0.001	-	0.001
MVPA	r	-0.510	0.337	-
	P	<0.001	<0.001	-

SED: sedentary time, LPA: light physical activity; MVPA: moderate-to-vigorous physical activity

Table S2. Bivariate correlations between PA, SED and GMV in the left and right hippocampi for the whole sample and stratified by weight status.

		All		Overweight		Obesity type I		Obesity type II-III	
		Left	Right	Left	Right	Left	Right	Left	Right
SED	r	0.046	-0.009	-0.151	-0.091	0.084	-0.032	0.212	0.264
	P	0.659	0.931	0.492	0.678	0.605	0.841	0.269	0.166
LPA	r	-0.040	-0.064	0.054	0.169	-0.088	-0.117	-0.006	-0.176
	P	0.698	0.561	0.808	0.440	0.584	0.465	0.974	0.362
MVPA	r	-0.022	0.099	0.187	0.051	0.108	**0.335**	**-0.435**	-0.269
	P	0.837	0.344	0.393	0.817	0.501	**0.033**	**0.018**	0.159

SED: sedentary time, LPA: light physical activity; MVPA: moderate-to-vigorous physical activity
